# Transgenerational transmission of a stress-coping phenotype programmed by early-life stress in the Japanese quail

**DOI:** 10.1038/srep46125

**Published:** 2017-04-07

**Authors:** Cédric Zimmer, Maria Larriva, Neeltje J. Boogert, Karen A. Spencer

**Affiliations:** 1 School of Psychology and Neuroscience, University of St Andrews, South Street, St Andrews, KY16 9JP, UK; 2 Centre for Ecology and Conservation, College of Life and Environmental Sciences, University of Exeter, Cornwall Campus, Penryn, TR10 9EZ, UK

## Abstract

An interesting aspect of developmental programming is the existence of transgenerational effects that influence offspring characteristics and performance later in life. These transgenerational effects have been hypothesized to allow individuals to cope better with predictable environmental fluctuations and thus facilitate adaptation to changing environments. Here, we test for the first time how early-life stress drives developmental programming and transgenerational effects of maternal exposure to early-life stress on several phenotypic traits in their offspring in a functionally relevant context using a fully factorial design. We manipulated pre- and/or post-natal stress in both Japanese quail mothers and offspring and examined the consequences for several stress-related traits in the offspring generation. We show that pre-natal stress experienced by the mother did not simply affect offspring phenotype but resulted in the inheritance of the same stress-coping traits in the offspring across all phenotypic levels that we investigated, shaping neuroendocrine, physiological and behavioural traits. This may serve mothers to better prepare their offspring to cope with later environments where the same stressors are experienced.

Conditions experienced in early-life can shape individual phenotypes and lead to irreversible modifications that may have long-term consequences for health and wellbeing^1^,^2^,^3^. This organizational effect, known as developmental programming, is a well-conserved phenomenon across vertebrates. It is mediated by glucocorticoid (GC) stress hormones that are released after the activation of the hypothalamo-pituitary-adrenal (HPA) axis in response to a stressful event^2^,^4^,^5^,^6^. Much previous work suggests a potential negative fitness outcome from prolonged or repeated activation of the HPA axis during early development^2^,^7^,^8^. However, recent studies looking at the consequences of early-life stress across life-history stages and in ecologically and evolutionarily relevant contexts have shown that early-life stress could prime offspring to cope better under stressful conditions in later life^4^,^9^,^10^,^11^,^12^,^13^,^14^. However, early-life stress is likely to have both benefits and costs. Its potential adaptive outcome may depend on the context within which the phenotypic traits are measured, as well as the life-history stage, the environmental conditions and the generation^1^,^4^,^6^,^15^,^16^.

Transgenerational effects of early-life conditions are defined as the consequences on offspring phenotype of the conditions experienced during development by the parental generation. The potential adaptive benefit of transgenerational transmission of developmentally programmed traits is attracting increasing research interest^1^,^17^,^18^. In vertebrates, early-life conditions can have transgenerational effects on a wide range of traits such as neuroanatomy, hormone levels, body size and condition, behaviour, reproductive success and survival (reviewed in ref. 1). It has been suggested that these effects may drive adaptive phenotypic plasticity, creating responses that could promote individual adaptation to changing environments^1^,^4^,^19^. However, as we are only at the onset of understanding the trans-generational effects of early-life conditions, their ecological consequences remain evasive^1^. One issue with many studies to date is the lack of a full factorial approach where both parental and offspring developmental environments are manipulated. Additionally, previous studies have usually focussed on a single phenotypic trait^1^,^18^,^20^. Finally, the potential consequences of stress interacting at different developmental stages to alter adult phenotypes in subsequent generations has not been investigated.

In this study, we manipulated pre- or/and post-natal stress in both mothers and their offspring in Japanese quail and examined the consequences for several phenotypic traits in the offspring generation, integrating information across physiological, neuroendocrine and behavioural levels. In all offspring, we determined the HPA axis activity by measuring the physiological response (i.e. changes in blood glucocorticoid (GC) levels) to an acute stressor. This stress response enhances physiological processes and behaviours to remove the individual from the stressor and/or facilitate coping. Although this acute response is adaptive, prolonged or repeated exposure to high GC levels can be costly over the long term^21^. The physiological stress response is tightly regulated by a negative feedback loop at the level of the hippocampus, hypothalamus and pituitary gland to down-regulate the HPA axis, which is mediated by the glucocorticoid receptor (GR) and the mineralocorticoid receptor (MR)^21^,^22^. We therefore measured the relative expression of both receptors within the HPA axis. At the behavioural level, we measured the exploration of a novel environment. An increased exploration of a novel environment is usually associated with a better capacity to cope with stressful challenges^23^. We used four groups of Japanese quail (*Coturnix japonica*) mothers with different developmental experiences: one of the groups was exposed to stress during pre-natal development via injection of corticosterone (CORT) in the egg yolk to simulate the transfer of CORT from the mother into her eggs (n = 11), one group was exposed to stress during post-natal development by exposing chicks to an unpredictable food availability paradigm simulating a stressful foraging environment (n = 6), one group was exposed to stress during both pre- and post-natal development (n = 6) and one control group (n = 10) was exposed to neither stressor^6^,^24^. The offspring of these females were directly exposed to stress during pre-natal development by manipulating maternal stress levels during the laying period using an unpredictable food availability paradigm (n = 45), or to control conditions (n = 42). Half of the chicks from each pre-natal treatment were exposed to stress using again an unpredictable food availability paradigm (n = 44) while the other half were exposed to control conditions during post-natal development (n = 43) (Fig. 1). This experimental design allowed us to determine the relative contribution of pre-natal and post-natal experiences and their potential interactive effects on offspring adult phenotype. It also allowed us to tease apart the effects of direct exposure of offspring to stress during their pre-natal (i.e. via manipulation of maternal stress), and/or post-natal development and the transgenerational effects of maternal exposure to early-life stress during their own pre- and/or post-natal development on shaping offspring phenotype.

## Results

### Stress response

Corticosterone (CORT) levels of offspring measured in adulthood were significantly influenced by the time upon capture by the experimenter (Table 1). Basal CORT (i.e. measured within 3 minutes of capture: 0.89 ± 0.07 ng.ml^−1^) was lower than CORT levels at 10 (2.65 ± 0.23 ng.ml^−1^) (t_71_ = 12.4, p < 0.0001) and at 30 (1.43 ± 0.13 ng.ml^−1^) minutes after capture (t_171_ = 6.0, p = 0.001) and the level at 10 minutes was higher than at 30 minutes after capture (t_170_ = 6.4, p < 0.001) (Fig. 2). This stress response over time was significantly influenced by maternal developmental experience at both developmental stages (maternal pre-natal treatment × maternal post-natal treatment × sampling time; Table 1). The multiple comparisons showed that this effect was mainly driven by maternal pre-natal treatment: for individuals whose mothers were exposed to pre-natal stress (MatPreCort), CORT concentrations significantly decreased between 10 and 30 minutes after capture (t_170_ ≥ 4.2, p ≤ 0.003; Fig. 2) and levels 30 min after capture did not significantly differ from baseline (MatPreCort) (t_172_ ≤ 2.0, p ≥ 0.69; Fig. 2) regardless of the maternal post-natal treatment. This decrease was not significant for individuals whose mothers were exposed to the pre-natal control treatment (MatPreCtrl) (t_170_ ≤ 2.3, p ≥ 0.48; Fig. 2), where CORT levels 30 min after capture remained significantly higher than basal levels (t_172_ ≥ 3.8, p ≤ 0.010; Fig. 2) regardless of the maternal post-natal treatment. However, offspring CORT levels 30 min after capture were not significantly different between individuals from mothers exposed to pre-natal stress (MatPreCort) or pre-natal control treatment (MatPreCtrl) regardless of maternal post-natal treatment (t_160_ ≤ 1.3, p ≥ 0.97; Fig. 2).

The offspring’s average CORT level of the 3 time points of the capture-handling restraint stress protocol were also significantly influenced by the interaction between both maternal treatments (maternal pre-natal treatment × maternal post-natal treatment) and by the interaction between both offspring treatments (offspring pre-natal treatment × offspring post-natal treatment) (Table 1). However, these two interactions are not functionally informative concerning the regulation of the physiological stress response as they do not concern the dynamic process by which CORT levels increase after capture to reach peak levels and the latency by which they return to baseline levels again. The magnitude of the peak and the latency to return to baseline level are known to have ecologically relevant consequences^21^,^25^ and are the outcome of the HPA axis feedback loop, and we therefore focus on those measures rather than CORT averages here.

### Novel environment

From all the measures we recorded in the novel environment, only the latency to enter the novel environment, the time spent in zone 3 of the novel environment (i.e. the area the furthest way from the introductory compartment and thus the ‘riskiest’) and the number of mealworms eaten were significantly influenced by one or more of the early-life treatments (Table 1).

The latency to enter the novel environment was only significantly influenced by the interactions between maternal pre-natal treatment and offspring post-natal treatment and, secondly, between maternal pre-natal treatment and offspring pre-natal treatment (Table 1). In both cases, this effect appeared to be mainly driven by the maternal pre-natal treatment: individuals whose mothers were exposed to pre-natal stress (MatPreCort) showed a significantly shorter latency to enter the novel environment compared to individuals whose mothers were in the pre-natal control group (MatPreCtrl) (z_85_ ≥ 2.71, p ≤ 0.034, Fig. 3a). This was the case regardless of the offspring’s own post-natal treatment (OffPostCtrl or OffPostFood-). Although multiple comparisons were not significant (z_85_ ≤ 2.44, p ≥ 0.07), the same trend was observed for offspring own pre-natal treatments, where maternal exposure to pre-natal stress tended to drive individuals to enter the novel environment faster (Fig. 3b).

The time spent in zone 3 of the novel environment was only affected by maternal pre-natal treatment and the interaction between both maternal treatments (Table 1). Offspring from pre-natally stressed mothers spent more time in zone 3 (MatPreCort: 48.7 ± 25.8 s), the part of the novel environment furthest away from the entrance, as compared to the offspring of pre-natal control mothers (MatPreCtrl: 32.6 ± 11.5 s; χ^2^_1,85_ = 4.82, p = 0.028). Offspring whose mothers had been exposed to both early-life stress treatments (MatPreCort/MatPostFood-) spent significantly more time in zone 3 than individuals whose mothers had been exposed to only one or no early-life stress treatments (z_85_ ≥ 2.0, p ≤ 0.046, Fig. 4).

Finally, the number of mealworms eaten in the novel environment was only significantly affected by the offspring post-natal treatment and the interaction between both maternal treatments (Table 1). Offspring exposed to post-natal food removal found and consumed more mealworms in the novel environment (OffPostCtrl: 0.6 ± 0.22, OffPostFood-: 0.91 ± 0.32 mealworms; χ^2^_1,85_ = 6.52, p = 0.011). Offspring from mothers exposed to both early-life stresses (MatPreCort/MatPostFood-) got more mealworms than those from mothers exposed to only one or none of the early-life stresses (z_85_ ≥ 2.58, p ≤ 0.05; Fig. 5).

### Glucocorticoid receptor and mineralocorticoid receptor mRNA expression

#### Hippocampus

In the offspring hippocampus, relative expression of the glucocorticoid receptor (GR) was only significantly influenced by maternal pre-natal treatment, whereas the relative expression of the mineralocorticoid receptor (MR) was not significantly impacted by any of the independent variables (Table 1). Offspring whose mothers had been exposed to pre-natal corticosterone had significantly increased GR mRNA expression (MatPreCort: 0.0095 ± 0.0015) as compared to individuals whose mothers were pre-natal controls (MatPreCtrl: 0.0045 ± 0.0007).

Due to the key role of GR and MR in the regulation of the stress response, the ratio between both receptors is crucial for stress resilience and homeostasis^22^,^26^. The GR:MR mRNA ratio was significantly higher in offspring exposed to pre-natal stress (OffPreFood-: 2.11 ± 0.79) compared to controls (OffPreCtrl: 1.02 ± 0.21) (Table 1). This ratio was also higher in the hippocampus of individuals whose mothers had been pre-natally stressed (MatPreCort: 2.15 ± 0.78) compared to individuals whose mothers were pre-natal controls (MatPreCtrl: 1.05 ± 0.36) (Table 1).

#### Hypothalamus

In the offspring hypothalamus, both GR and MR relative expression was only significantly influenced by maternal pre-natal treatment and offspring pre-natal treatment whereas the GR:MR mRNA ratio was not affected by any of the independent variables (Table 1). For both receptors, pre-natal stress (MatPreCort and OffPreFood-) was associated with an increased mRNA expression (Fig. 6a,b).

#### Pituitary gland

In the offspring pituitary gland, GR and MR mRNA levels were only significantly impacted by maternal pre-natal treatment whereas the GR:MR mRNA ratio was not affected by any of the independent variables (Table 1). Glucocorticoid and mineralocorticoid receptor mRNA expression was higher in offspring whose mothers had been prenatally stressed (MatPreCort, GR: 0.0303 ± 0.0044; MR: 0.0233 ± 0.0076) compared to individual whose mothers were pre-natal controls (MatPreCtrl, GR: 0.0186 ± 0.0025; MR: 0.0102 ± 0.0015).

## Discussion

By using a full factorial experimental design where both maternal and offspring early-life experiences were manipulated, we showed that maternal stress experienced during their early-life had a stronger effect on offspring adult phenotype than the offspring’s own early-life experience at all phenotypic levels that we investigated, shaping offspring neuroendocrine, physiological and behavioural traits. Our previous studies show that pre-natal stress has the power to engineer a “stress-coping phenotype” in the maternal generation^14^,^24^. Here, maternal pre-natal experience did not simply alter offspring phenotype but resulted in the transmission of this maternal “stress-coping phenotype”. This may allow mothers to better prepare their offspring to face stressful situations in their future environment. Our results also highlight the relative strength of pre-natal compared to post-natal experiences, as most of the long-term phenotypic effects resulted from manipulation of pre-natal experience whereas post-natal conditions only affected offspring behaviour but not neuro-endocrine traits.

At the neuroendocrine level, offspring of pre-natally stressed females exhibited an increased GR mRNA expression and GR:MR mRNA ratio in the hippocampus and higher GR and MR mRNA levels in the hypothalamus and in the pituitary gland compared to individuals of pre-natal control mothers. Offspring early-life experience also affected receptor expression in the HPA axis: offspring pre-natally stressed showed an increased GR:MR mRNA ratio in the hippocampus and GR and MR mRNA level in the hypothalamus. These modifications are in accordance with the effects of pre-natal stress observed in the maternal generation: pre-natally stressed mothers showed a larger GR:MR mRNA ratio in the hippocampus, an increased GR and MR mRNA expression in the hypothalamus and a higher GR mRNA expression in the pituitary gland^14^. Hence, it appears that maternal exposure to pre-natal stress resulted in the same intracellular receptor expression profiles in the HPA axis in both generations, regardless of whether the offspring were exposed to pre-natal stress themselves. In contrast, other changes in offspring phenotype induced by direct exposure to pre-natal stress were not exactly the same, and often weaker, than those induced by maternal pre-natal stress exposure. The GR and MR receptors play a crucial role within the HPA axis for the regulation of glucocorticoids at baseline and stress-induced levels and in the negative feedback efficiency^22^,^26^,^27^. In the maternal generation, these modifications of GR and MR mRNA expression within the HPA axis in pre-natally stressed individuals resulted in a more efficient negative feedback and thus were associated with an attenuated stress response to an acute stressor^14^,^24^. Similarly, here we show that corticosterone levels in the offspring of pre-natally stressed mothers decreased by more than 50% from 10 to 30 minutes after capture and returned to a lower level not significantly different from baseline level, whereas it decreased by only ca. 40% and remained significantly higher than baseline level in the offspring of pre-natal control females. Despite these different physiological dynamics, corticosterone levels at 30 minutes were not significantly different between offspring of pre-natal stressed and control females. Maternal exposure to pre-natal stress thus resulted in an attenuated physiological stress response in their offspring, which we suggest is mediated by the same neuroendocrine changes in the HPA axis in both generations. However, direct application of pre-natal stress to offspring did not result in an attenuated physiological stress response despite its effect on GR and MR mRNA expression within the HPA axis. This suggests that these modifications of receptors were not strong enough to affect the metrics of the physiological stress response that we measured. One potential caveat is that we measured GR and MR mRNA levels but we did not determine GR and MR protein expression. Some recent studies suggest that mRNA and protein expression measures do not always correlate (e.g refs 28 and 29), although most studies show that they are coupled^30^,^31^,^32^,^33^,^34^,^35^,^36^. The observed changes in GR and MR mRNA expression in our study are in accordance with the mediation of the physiological acute stress response, but it would be interesting to directly measure whether GR and MR mRNA and protein levels are coupled in the Japanese quail.

At the behavioural level, we employed the same novel environment test in our assessment of exploration in both generations. Offspring of females exposed to pre-natal stress took less time to enter the novel environment and spent more time away from the introductory compartment (in Zone 3) compared to offspring of pre-natal control females. Thus, maternal exposure to pre-natal stress induced an increase in exploration behaviour in a stressful novel environment in their offspring. In accordance with the results in the maternal generation^24^ and in other mammal and bird species^37^,^38^,^39^,^40^, we suggest that this increased exploration was mediated by the attenuated physiological stress response programmed by maternal exposure to pre-natal stress. In addition, maternal exposure to both pre- and post-natal stress had interactive effects on offspring behaviour, where these individuals spent more time away from the introductory compartment (in Zone 3) and ate more mealworms than offspring of females that were exposed to none or only one early-life stressor. These are the exact same behavioural responses as we observed in the maternal cohort when tested at the same age as their offspring^24^. We therefore suggest that the phenotype programmed by exposure to early-life stress is readily transmitted to the following generation. Offspring direct exposure to post-natal stress was associated with a higher consumption of mealworms in the novel environment. This result is in accordance with previous studies showing that post-natal stress increases risk-taking and motivation to find food later in life^24^,^38^,^41^,^42^,^43^,^44^.

It appears that pre-natal stress (either maternal or offspring direct exposure) affected the offspring phenotype more than post-natal stress (either maternal or offspring direct exposure). This is in agreement with previous studies in the Japanese quail and others species showing that the pre-natal period is a sensitive period to stress that could result in long-term impacts on the phenotype (human and rodents^2^,^7^,^8^,^45^, guinea pig^46^, birds^47^, chicken^48^,^49^, Japanese quail^6^,^14^,^24^,^50^, threespine stickleback^10^,^51^, guppies^52^). However, in some other species post-natal experience does have a strong effect on individual phenotype (rat^9^,^15^,^53^,^54^, zebra finch^44^,^55^,^56^,^57^,^58^,^59^). It seems that the main difference between the species that are more likely affected by pre-natal or by post-natal stress is the degree of development at birth. In precocial species such as the Japanese quail, chicken or hare, pre-natal stress appears to have the strongest effect on adult phenotype whereas in altricial species like the zebra finch or rat, it seems that post-natal stress is the main factor affecting the adult phenotype.

Similarly to recent findings in the zebra finch^18^ where the authors manipulated both parent and offspring early nutritional conditions, we did not observe any interactive effects between maternal and offspring developmental experiences where both affected the traits we measured. We manipulated both maternal and offspring pre-natal experience and we showed that maternal pre-natal stress had a more substantial impact on offspring phenotype later in life regardless of the offspring’s own direct exposure to stress during development. Both maternal and offspring direct exposure to pre-natal stress independently affected GR and MR mRNA levels in the HPA axis. However, the effect of the offspring’s exposure to pre-natal stress was weaker than the effect of the maternal pre-natal experience. Moreover, contrary to what was observed in the maternal generation^24^, direct application of pre-natal stress to offspring did not result in an attenuated physiological response and neither had it effects on behaviour in the novel environment.

We showed here that maternal pre-natal stress exposure had a stronger effect in shaping offspring phenotype than their own pre-natal stress exposure. This might be because we exposed the maternal and offspring generations to different pre-natal stressors: corticosterone injection into the egg for the mothers *vs*. random food removal during the maternal formation of the eggs from which the test offspring emerged. The amount of corticosterone we injected in the eggs to produce the maternal pre-natal stress treatment group elevated yolk corticosterone in the physiological range (1.8 SD above the lab population average). However, the random food removal during the mothers’ egg laying period may not have affected maternal corticosterone deposition into the eggs, or not to an extent equivalent to the corticosterone injection in the maternal generation. Consequently offspring may not have been exposed to the same pre-natal stress level as the maternal generation, which would explain the strong effect of maternal pre-natal stress and the weak effects of offspring pre-natal stress. We suggest that future studies quantify the corticosterone levels in the eggs laid by females in the food-removal treatment group and the control group to determine the efficiency of the food-removal treatment to manipulate offspring pre-natal corticosterone exposure. Nevertheless, this suggests that different stressors can result in distinct effects on the phenotype^46^.

It is now well recognised that stress exposure during early development may not only affect individuals directly exposed to the stress, but also their offspring and grand-offspring through transgenerational effects^1^,^7^,^17^. Nevertheless, it is important to make the distinction between transgenerational *effects* and transgenerational *transmission*. Transgenerational effects occur when early developmental conditions in one generation affect future generations but not necessarily in the same way. Transgenerational transmission or inheritance refers to the transmission of the same modification in one or more phenotypic traits induced by early developmental experiences from one generation to the next. Examples of transgenerational effects of early-life conditions are quite abundant, especially in the biomedical, epidemiological and toxicological literature on humans and rodents (reviewed in refs 1 and 17). Studies showing transgenerational inheritance of phenotypic traits programmed by early-life conditions are more limited^60^,^61^ (but see rodents^53^,^60^,^62^,^63^,^64^; birds^50^,^65^,^66^). However, most of these studies have shown transgenerational transmission at a single phenotypic level. An exception is the research on rats where the transmission of the maternal licking and grooming (LG) behaviours is associated with the transmission of an attenuated response to stress mediated by an increase in the expression of GR in the hippocampus and of a reduced behavioural response to fearful stimuli^53^,^63^. Our results show transgenerational transmission of a suite of linked phenotypic traits from the hormone receptor to the behavioural level programmed by maternal exposure to pre-natal stress. It has been proposed that the physiological and behavioural changes we observed may help to cope better with stressful situations and thus may have positive consequences for fitness^23^,^67^,^68^,^69^. Therefore, it appears that mother’s “stress-coping phenotype” programmed by early-life stress can be transmitted to the next generation which may enhance their offspring’s capacity to cope with later environments that vary in resources or in biotic and abiotic factors leading to the activation of the HPA axis.

The transmission of the maternal stress-coping phenotype to their offspring could be mediated by two non-exclusive non-genomic transmission pathways. Firstly, pre-natally stressed females may have altered the pre-natal environment of their offspring^17^ by increasing the amount of corticosterone deposited into the eggs. This would directly expose developing chicks to increased pre-natal stress hormone levels that may have programmed their phenotype in the same way as that observed in the maternal generation. Another potential mechanism is transmission via alteration of the epigenome^1^,^17^. In the example of licking/grooming behaviour in rat dams, a high level of these behaviours from mothers towards their pups altered the pups’ epigenome at the level of the GR promoter in the hippocampus. This epigenetic modification increased GR expression in the hippocampus and lead to the observed attenuated physiological stress responses and increased L/G behaviour of pups^70^. Interestingly, pre-natal stress exposure in the maternal generation in this quail population also resulted in modifications of GR and MR mRNA expression in the HPA axis, causing the observed attenuated stress response and behavioural modification in the novel environment test^14^,^24^. As early life seems to be a sensitive period for the environment to affect the epigenome^1^, we can hypothesize that the differential receptors’ expression may result from epigenetic alterations of the GR and MR genes due to exposure to pre-natal stress. If these epigenetic modifications were inherited by the offspring it would explain the development of the same phenotype. It would therefore be interesting to determine if pre-natal stress affected CORT deposition into the egg and/or epigenetic status of GR and MR genes in order to determine the causal mechanism underlying the transgenerational transmission of the maternal phenotype in more detail.

Our exhaustive experimental design in which both mothers and their offspring’s developmental conditions were manipulated allowed us to tease apart ancestral and current modulators of a range of phenotypic traits. We show that maternal pre-natal experience was the main driver of their offspring’s phenotype expression, resulting in the offspring inheritance of their mother’s ‘stress-coping phenotype’ programmed by pre-natal stress. This may allow mothers to enhance their offspring’s capacities to cope with a stressful environment later in life, which ultimately may increase their fitness. Such transgenerational non-genetic inheritance can be adaptive in changing environments when environmental fluctuations are predictable and the information provided by the parents are reliable. Under those circumstances, parents will benefit from transmitting their phenotypes optimized for anticipated conditions^19^,^71^,^72^. These results also support the notion that developmental programming should be regarded as a transgenerational phenomenon^17^ that could be crucial for adaption to changing environments.

## Methods

### Maternal stress manipulations

**Figure 1:**
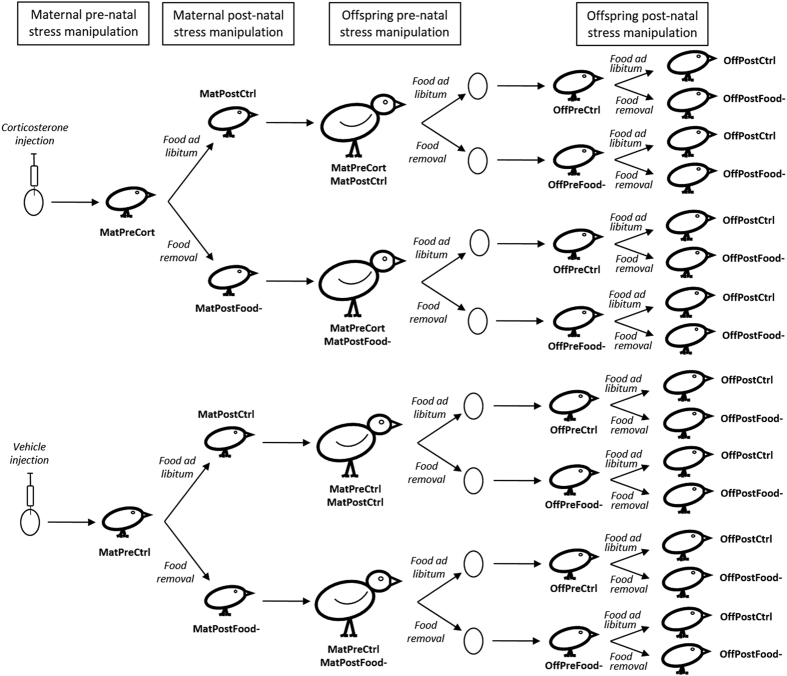
Diagram of the experimental manipulation of maternal and offspring pre-natal and post-natal stress. We used four groups of Japanese quail females with different developmental experiences in order to obtain the offspring generation. One group was exposed to stress only during pre-natal development via injection of corticosterone in the egg yolk to simulate the transfer of CORT from the mother into her eggs (MatPreCort/MatPostCtrl, n = 11). One group was exposed to stress only during post-natal development by exposing chicks to unpredictable food availability between days 4 and 20 post-hatching (MatPreCtrl/MatPostFood-, n = 6). One group was exposed to stress during both pre- and post-natal development (MatPreCort/Mat/PostFood-, n = 6). The control group was exposed to neither stressor (MatPreCtrl/MatPostCtrl, n = 10). Each female bred once under control condition and once under unpredictable food availability in order to manipulate pre-natal stress of the offspring by increasing maternal stress levels during the laying period (Offspring pre-natal stress manipulation: pre-natally stressed n = 45, control n = 42). Half of the chicks from each pre-natal treatment were exposed to stress again using an unpredictable food availability paradigm (n = 44) while the other half were exposed to control conditions during post-natal development (n = 43). As for the offspring, we thus created four treatment groups: OffPreCtrl/OffPostCtrl (n = 22); OffPreCtrl/OffPostFood- (n = 20), OffPreFood-/OffPostCtrl (n = 21) and OffPreFood-/OffPostFood- (n = 24). This experimental design resulted in 16 treatment combinations.

**Figure 2:**
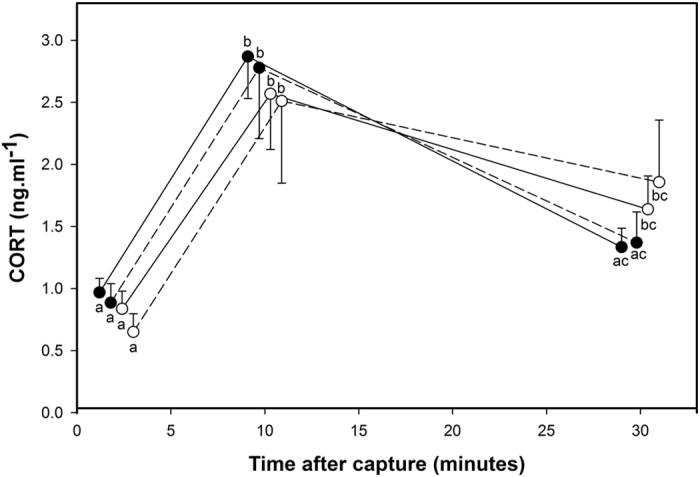
Maternal exposure to pre-natal stress resulted in an attenuated stress response in their offspring. CORT level modification in response to a capture-handling-restraint protocol in offspring of mothers exposed to pre- and post-natal control treatments (white dots and solid line), of mothers exposed to pre-natal control and post-natal stress treatments (white dots and dashed line), of mothers exposed to pre-natal stress and post-natal control treatments (black dots and solid line) and of mothers exposed to pre- and post-natal stress treatments (black dots and dashed line). Values are means ± SEM. Different letters indicate significant differences.

**Figure 3:**
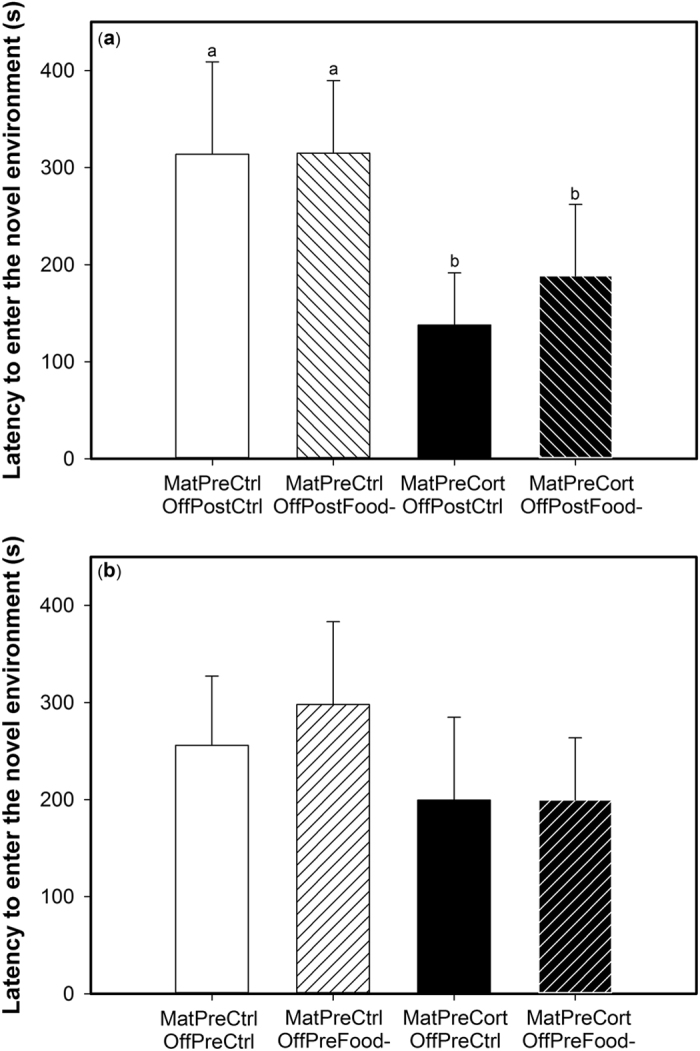
Maternal exposure to pre-natal stress decreased their offspring’s latency to enter the novel environment. Mean latency in seconds to enter the novel environment, as affected by (**a**) the interaction between maternal pre-natal treatment (control: MatPreCtrl, white bars, stressed: MatPreCort, black bars) and offspring post-natal treatment (control: OffPostCtrl, empty bars, stressed: OffPostFood-, right hatched bars) and (**b**) the interaction between maternal pre-natal treatment (control: MatPreCtrl, white bars, stressed: MatPreCort, black bars) and offspring pre-natal treatment (control: OffPreCtrl, empty bars, stressed: OffPreFood-, left hatched bars). Values are means ± SEM. Different letters indicate significant differences.

**Figure 4:**
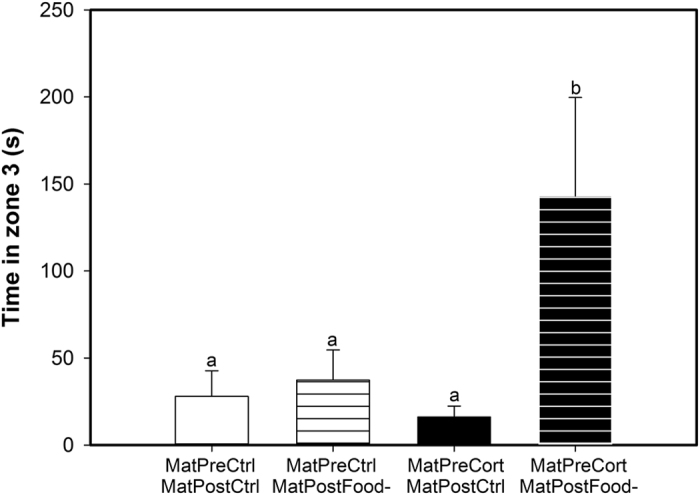
Maternal exposure to both early life stressors increased the time their offspring spent away from the home cage in the novel environment. Mean time spent in zone 3 for the offspring of mothers exposed to pre- and post-natal control treatments (MatPreCtrl/MatPostCtrl, empty white bar), of mothers exposed to pre-natal control and post-natal stress treatments (MatPreCtrl/MatPostFood-, striped white bar), of mothers exposed to pre-natal stress and post-natal control treatments (MatPreCort/MatPostCtrl, empty black bar) and of mothers exposed to both pre- and post-natal stress treatments (MatPreCort/MatPostFood-, striped black bar). Values are means ± SEM. Different letters indicate significant differences.

**Figure 5:**
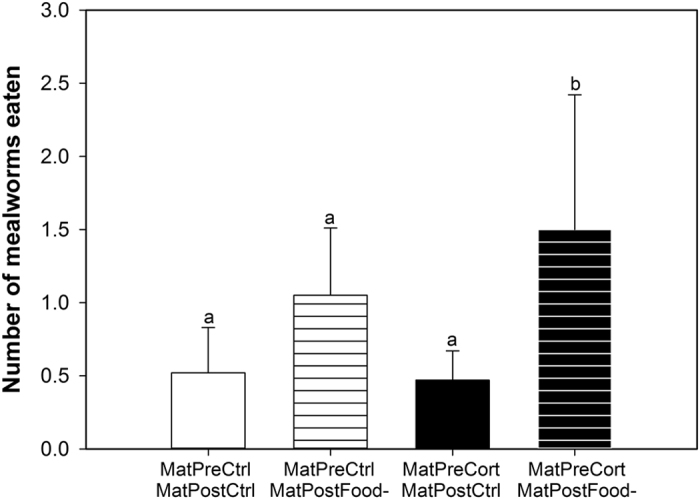
Maternal exposure to both early life stressors increased the number of mealworms their offspring ate in the novel environment. Mean number of mealworms eaten for the offspring of mothers exposed to pre- and post-natal control treatments (MatPreCtrl/MatPostCtrl, empty white bar), of mothers exposed to pre-natal control and post-natal stress treatments (MatPreCtrl/MatPostFood-, striped white bar), of mothers exposed to pre-natal stress and post-natal control treatments (MatPreCort/MatPostCtrl, empty black bar) and of mothers exposed to pre- and post-natal stress treatments (MatPreCort/MatPostFood-, striped black bar). Values are means ± SEM. Different letters indicate significant differences.

**Figure 6:**
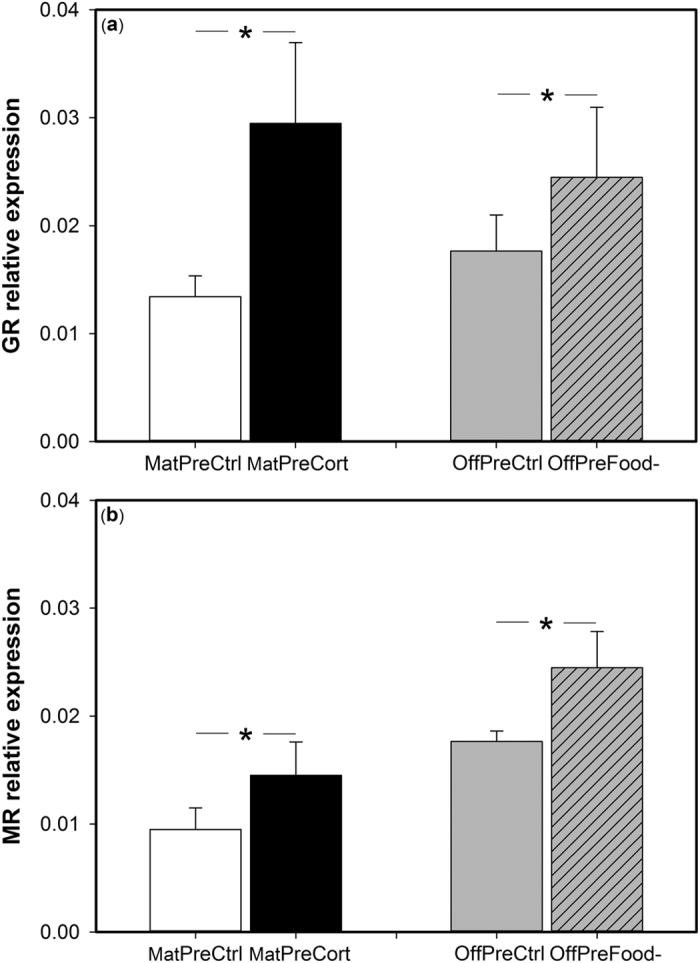
Maternal exposure and offspring exposure to pre-natal stress increased offspring glucocorticoid and mineralocorticoid receptor expression in the hypothalamus. Relative expression of (**a**) glucocorticoid receptor mRNA (GR) in the hypothalamus of offspring of pre-natal control mothers (MatPreCtrl, white bar) and of pre-natally stressed mothers (MatPreCort, black bar) and in pre-natal control offspring (OffPreCtrl, empty grey bar) and in pre-natally stressed offspring (OffPreCort, left hatched grey bar) (**b**) mineralocorticoid receptor mRNA (MR) in the hypothalamus of offspring of pre-natal control mothers (MatPreCtrl, white bar) and of pre-natally stressed mothers (MatPreCort, black bar) and in pre-natal control offspring (OffPreCtrl, empty grey bar) and in pre-natally stressed offspring (OffPreCort, left hatched grey bar). Values are means ± SEM. *Indicates significant differences.

All of the procedures carried out in this study were approved by the local ethics committee at the University of St Andrews (Scotland) and the experiment was conducted in accordance with the Animals (Scientific Procedures) Act 1986 (under PIL 70/13261 and PIL 70/1364 held by CZ and KAS respectively and PPL 60/4068 held by KAS). 76 unrelated fertile Japanese quail eggs were incubated. After 5 days of incubation, we manipulated pre-natal stress in half of these eggs by injecting them with 10 μl of corticosterone (CORT) dissolved in sterile peanut oil (850 ng.ml^−1^) at the egg apex under sterile conditions (MatPreCort: n = 38). This dose is known to increase yolk CORT concentrations within 1.8 SD of those found in the control breeding population^24^,^41^ and similar to previous studies that have increased CORT within the physiologically relevant range or used natural stressors to increase CORT deposition into the eggs (eg. refs 13, 73, 74, 75). Control eggs were injected with peanut oil alone (MatPreCtrl: n = 38) (Fig. 1). At hatching, chicks (n = 59) were individually marked with a unique pattern of colors using nail polish to allow individual recognition. Chicks of each pre-natal treatment were kept in two different enclosures with *ad libitum* food. When they were 4 days old, half of the chicks of each pre-natal treatment were assigned to one of two post-natal food treatments: either food removal on a random schedule for 3.5 h per day (25% of daylight hours) between 8 A.M. and 8 P.M. between the age of 4–20 days (MatPostFood-: n = 28) or *ad libitum* food at all times during the same period (MatPostCtrl: n = 31) (Fig. 1, see refs 24 and 41 for details). After the end of the post-natal treatment, all birds were provided with *ad libitum* food until adulthood and the breeding experiments began. We thus created four maternal treatment groups: MatPreCtrl/MatPostCtrl (n = 15, 10 females); MatPreCtrl/MatPostFood- (n = 13, 6 females), MatPreCort/MatPostCtrl (n = 16, 11 females) and MatPreCort/MatPostFood- (n = 15, 6 females) (Fig. 1). From this cohort, the 33 females were used to produce offspring in order to look at potential transgenerational effects.

### Offspring stress manipulations

During breeding of the maternal generation, females were placed in individual cages (76 × 48 × 53 cm) and a control male was placed in each female cage for 10 minutes once a day. This has been shown to be an effective way to produce fertile eggs whilst minimizing harassment of females from males^76^. We used eight different control males, each male was presented to four females every day. The order of presentation of females was randomly assigned every day for each male. Each female produced two clutches 8 weeks apart under two different environmental conditions in order to match or mismatch their adult environment with their developmental environment and to manipulate offspring pre-natal stress. One condition was designed to mimic unpredictable food stress, and elevate maternal CORT levels without causing food restriction^77^,^78^. Food was randomly removed for 25% of the daylight hours (3.5 h) every day for 28 days between 0800 h and 2000 h during the laying period (OffPreFood-). The second condition was a control treatment where females were provided with *ad libitum* food access throughout the laying period (OffPreCtrl). Both treatments (stress: OffPreFood- and control: OffPreCtrl) and the order of each treatment were counterbalanced in each female group to control for any effect of the first clutch treatment on the response for the second clutch (Fig. 1). For each clutch, eggs laid during the first 10 days of mating were removed as this is the minimum time required to obtain fertile eggs^79^. Consequently cages were checked every day and each egg was given a number in order to track laying order. Random food removal has been shown to increase peak CORT levels^77^ and 7 days is the minimum time required for an increase in plasma CORT to result in an increase in CORT level in the yolk in the Japanese quail^80^. Thus, to increase the probability of a transfer of a higher CORT volumes into the eggs, for each female the 2 eggs laid on the last 2 days of the breeding treatment were collected in both conditions and were incubated. If a female finished her clutch before the end of the treatment the last 2 eggs laid were incubated. Consequently, for each female, 2 eggs produced under stress conditions and 2 eggs produced under control conditions were incubated. Two females under control breeding conditions did not lay a clutch. For 3 females reproducing under control conditions and 4 reproducing under stress conditions, eggs were not fertile. Consequently, 114 eggs (OffPreCtrl: n = 58; OffPreFood-: n = 56) were incubated at 37.5 °C and 55% humidity. On day 14 of incubation, eggs were moved to hatchers where eggs of each female were kept in different compartments, where they were maintained at 37 °C and 75% humidity until hatching on day 18. Eighty-seven eggs hatched (OffPreCtrl: 77.6%, OffPreFood-: 80.4%). Upon hatching, each chick was given a unique nail polish mark on the head and wings and was returned to the hatcher for 24 hours to allow feathers to dry. Chicks of each pre-natal treatment were kept in two different enclosures with *ad libitum* food, water and an electric hen brooder. When chicks were 4 days old, if the two chicks of a female hatched each chick was subsequently randomly allocated to one of the two post-natal treatments: either food removal on a random schedule for 3.5 h per day (25% of daylight hours) between 0800 and 2000 h between the age of 4–20 days (OffPostFood-: n = 44) or *ad libitum* food during the same period (OffPostCtrl: n = 43) (Fig. 1). If only one chick hatched for a female, the chick was allocated to one of the two post-natal treatments to obtain an equal number of chicks from the two pre-natal conditions in each group. Room temperature was maintained at 20–22 °C and the photoperiod was 14L:10D (0700 h–2100 h). At 20 days post-hatching, all birds were provided with access to *ad libitum* food. We thus created four offspring treatment groups: OffPreCtrl/OffPostCtrl (n = 22); OffPreCtrl/OffPostFood- (n = 20), OffPreFood-/OffPostCtrl (n = 21) and OffPreFood-/OffPostFood- (n = 24). This experimental design resulted in 16 treatment combinations (Fig. 1) allowing us to determine if offspring phenotype was shaped by direct exposure to early life-stress, by exposure of their mother to early-life stress, by both direct and maternal exposure to early-life stress or if early-life stress had no effects on offspring phenotype. These treatment groups also gave us the opportunity to assess at which developmental stage early-life stress altered adult phenotype and if stress exposure at different developmental stages had interactive effects on offspring phenotype.

### Physiological stress response

Physiological stress response was assessed using a standardized capture-handling-restraint stress protocol when offspring were between 40 and 45 days of age (43 ± 0.2 days). One week before the beginning of blood sampling, birds were moved to individual cages (76 × 48 × 53 cm) in two rooms in which both sides of the rooms were visually divided so birds could not see the experimenters entering. Consequently, we were able to catch birds from one side of the room without disturbing birds located at the other side. When birds had been caught on both sides of the room, we waited 2 hours before catching birds again in the same room to avoid any effects of the previous captures. Additionally, catching order was added as a covariate in the analyses and showed no effect. Between 0900 and 1200 h, three experimenters silently entered a room and each caught a quail in its cage and then went to a nearby room where the blood was collected. Blood (70 μl) was collected within 3 minutes of capture to determine baseline CORT levels. Birds were then placed in an opaque box and two more blood samples were collected 10 and 30 minutes after initial capture. Samples were taken by venipuncture of a brachial vein. Blood was collected in a heparinised capillary tube and then transferred into a microcapillary tube and kept on ice until centrifugation (within 3 hours). Samples were centrifuged for 10 minutes at 3500 rpm and plasma stored at −20 °C for later analysis.

CORT concentrations were measured after extraction of 30 μl aliquots of plasma in 1 ml of diethyl ether by the dextran-coated charcoal radioimmunoassay method (DextranT70, Sigma Aldrich 31390-25G, Activated Charcoal, Acros Organics 134342500), using CORT antiserum code ABIN344880 (antibodies-online.com) and [1,2,6,7-3H]-CORT label. The cross-reactivity of the antibody is <0.01–1.5%, depending on the compound. CORT levels of all birds were above the detection limit (0.08 ng.ml^−1^). For all samples, extraction efficiency was estimated and ranged between 69 and 100%. All samples were run in duplicate in three assays and intra-assay and inter-assay coefficients of variation were 0.06 and 0.09, respectively. CORT concentration at 50% binding was 1.03 ng.ml^−1^. All samples from a single individual were quantified in the same assay and treatment groups were equally represented within each assay.

### Novel environment exploration

When offspring were between 50 and 59 days of age (55.1 ± 0.3 days), exploratory behaviour in a novel environment was measured according to the same protocols used for mothers^21^. The novel environment was a cage (120 × 75 × 75 cm) containing an introductory compartment (25 × 25 cm) and four novel objects: 2 orange plastic traffic cones (17.5 cm high × 10 cm wide at the base), a multi-coloured enclosed feeder made of plastic brick (26 cm × 19 cm × 15 cm), a cloth ring with coloured fabrics (28 cm high × 30 cm wide) and two yellow opaque plastic panels, one of which hid one of the traffic cones while the other panel hid the cloth ring with coloured fabrics. The second traffic cone was positioned close to the exit of the introductory compartment. Three small white opaque plastic dishes (7 cm diameter × 4 cm high) containing three dried mealworms each were placed near both hidden objects and in the plastic brick construction (see ref. 24 for details). After 80 minutes of food deprivation in their individual cages, birds were moved to the introductory compartment of the novel environment and allowed to habituate for another 10 minutes without food. Then, the experimenter opened a sliding door to allow the test subject to enter the novel environment and left the room. The sliding door remained open during the duration of the test allowing the individual to come back in the introductory compartment. The behaviour of the test subject was recorded for 15 minutes with two camcorders: one in front of the cage and one above the cage. For video analyses, we imagined the cage to be divided into three equally sized zones (see ref. 24 for details). From the videos, we recorded the latency to exit the introductory cage, the latency to enter each of the three virtual zones, latency to feed from each of the three feeders, the time spent in each zone and in the introductory compartment, the number of feeders visited, the number of mealworms eaten and the time spent moving.

### Tissue collection and quantitative real-time PCR

At the end of the experiment, when individuals of the offspring generation were 73.2 ± 0.5 days old, they were sacrificed by injection of an overdose of pentobarbital. Brains were quickly removed (within 1 minute), then pituitary glands were also removed (within 1 minute after brain removal) and placed on dry ice until frozen, then stored at −80 °C. To perform the dissections, the brains were placed ventral side up into a brain Matrix with a 1 mm graduated scale placed on a mixture of dry and wet ice to keep the brain frozen and a 2 mm-thick coronal section was cut using two razor blades. Then, whilst still frozen, two equivalent bilateral punches (1 mm diameter each) were obtained from the hippocampus and a single punch was obtained from the medial hypothalamus that spanned the third ventricle (see ref. 14 for details). Each sample was stored separately at −80 °C.

Total RNA was extracted and purified using Absolutely RNA Miniprep kits (Agilent Technologies, Santa Clara, CA, USA) according to the manufacturer’s protocol. The quantity and integrity of RNA were assessed with a RNA 6000 Pico assay kit for hippocampus and hypothalamus and a RNA 6000 Nano kit for pituitary gland using the Agilent 2100 bioanalyzer according to the manufacturer’s instructions. The mean RNA integrity number (RIN) of these samples was 7.6 ± 0.1 (range: 4.5–9.5). First strand cDNA was synthesized using Affinity Script Multiple Temperature cDNA Synthesis kits (Agilent Technologies) and diluted to obtain a working concentration of 25 pg.μl^−1^. This cDNA was used to perform quantitative real-time PCR (qPCR) for the genes of interest (GR and MR) and the house-keeping gene β-actin (BA) for the different brain regions using gene-specific primers. We used Specific PerfectProbe™ primers (Primerdesign, Southampton, UK) for the genes of interest that amplified single products with no dimer pairs and have been validated in our quail population as well as the house keeping gene using a chicken (*Gallus gallus*) Genorm kit (Primerdesign) (BA: M = 0.30; other candidates M ≥ 0.34)^14^. GR sense primer: TAATGACCGTGGTGACCTTTTA, anti-sense primer: TTTCTTGCTTTATGCCAGGAGTA (GenBank accession no. NM_001037826). MR sense primer: GTAGAATAGAGGACAGATGAACTTTT, anti-sense primer: ACCCAGAGAGAACACTACAGAT (GenBank accession no. NM_001159345). All qPCR reactions were run in duplicate with all the samples of an individual on the same plate. Each reaction contained 10 μl of 2x Brilliant III Ultra-Fast QPCR Master Mix (Agilent technologies), 1 μl of specific PerfectProbe™ primer at a working concentration of 300 nM, 0.3 μl of reference dye, 3.7 μl of RNAse/DNAase-free water and 5 μl of appropriate cDNA for a final volume of 20 μl along with no-template controls and blanks. Reactions were carried out on a Stratagene MX 3005 P (Agilent Technologies) at 95 °C for 3 min, then 50 cycles of 95 °C for 20 s, 60 °C for 20 s. From standard curves generated with known concentrations of cDNA, we determined that the amplification efficiency (Eff = 10^(−1/slope)^ − 1) was higher than 94% for GR, MR and BA. Therefore, we used the Delta Ct method (ΔCt) to quantify the relative expression of GR and MR mRNA relative to BA: 2^−(Ct GR/MR−Ct BA)^^81^.

### Statistical analyses

For all dependant variables, offspring early-life treatments were specified as fixed factors to look at potential programming effects of these treatments on phenotypic traits. Maternal early-life treatments were also added as fixed factors in the models to look at potential transgenerational effects. All models also included offspring sex and all the interactions between sex and the maternal and offspring treatments. For all models, residuals of normal models were not normally distributed, so generalized linear mixed models (GLMM) were used. Individual identity was used as a random factor to account for inter-individual differences. Mother identity was also specified as a random factor to take into account the parentage between individuals.

A GLMM was performed to analyse CORT levels. The model was fitted with a gamma law and the MSPL (Maximum Subject-specific Pseudo-Likelihood) was used as the estimation method. Blood-sample time after capture was also included as a repeated factor to take into account the non-independence between samples.

GLMMs fitted with a gamma law were used to analyse relative GR and MR mRNA expression and GR:MR mRNA ratio in the different brain regions; latencies to enter the novel environment and to feed in the three feeders; time spent in the introductory compartment, in the three zones and time spent moving in the novel environment. GLMMs fitted with Poisson law were used to analyse the number of feeders visited and the number of mealworms eaten.

GLMMs were performed using the GLIMMIX procedure in SAS 9.4. For all models, Tukey-Kramer multiple comparison adjustment was applied to obtain corrected p-values. Probability levels <0.05 were considered as significant. Data presented are means ± SEM.

## Additional Information

**How to cite this article**: Zimmer, C. *et al*. Transgenerational transmission of a stress-coping phenotype programmed by early-life stress in the Japanese quail. *Sci. Rep.* **7**, 46125; doi: 10.1038/srep46125 (2017).

**Publisher's note:** Springer Nature remains neutral with regard to jurisdictional claims in published maps and institutional affiliations.

## Figures and Tables

**Table 1:** Statistical results for all independent factors and second order interactions between them, and only for significant interactions of higher order for all the models.

Variables	Factors	DF	F/χ^2^-value	P-value
CORT levels	Maternal pre-natal treatment	1,85	2.47	0.120
	Maternal post-natal treatment	1,85	3.12	0.081
	Offspring pre-natal treatment	1,85	0.06	0.801
	Offspring post-natal treatment	1,85	0.83	0.370
	Sex	1,85	3.22	0.077
	Sampling time	2,170	77.13	<**0.0001**
	Maternal pre-natal treatment × maternal post-natal treatment	2,170	7.75	**0.007**
	Offspring pre-natal treatment × offspring post-natal treatment	1,85	6.31	**0.014**
	Maternal pre-natal treatment × sex	1,85	2.15	0.146
	Maternal post-natal treatment × sex	1,85	0.11	0.740
	Offspring pre-natal treatment × sex	1,85	0.15	0.700
	Offspring post-natal treatment × sex	1,85	3.93	0.052
	Maternal pre-natal treatment × offspring pre-natal treatment	1,85	0.62	0.434
	Maternal pre-natal treatment × offspring post-natal treatment	1,85	0.83	0.365
	Maternal post-natal treatment × offspring pre-natal treatment	1,85	1.72	0.193
	Maternal post-natal treatment × offspring post-natal treatment	1,85	0.58	0.450
	Maternal pre-natal treatment × sampling time	2,170	1.77	0.173
	Maternal post-natal treatment × sampling time	2,170	1.82	0.165
	Offspring pre-natal treatment × sampling time	2,170	0.05	0.948
	Offspring post-natal treatment × sampling time	2,170	0.01	0.994
	Maternal pre-natal treatment × maternal post-natal treatment × sampling time	2,170	6.83	<**0.0001**
Latency to enter the novel environment	Maternal pre-natal treatment	1,85	2.34	0.126
	Maternal post-natal treatment	1,85	3.54	0.061
	Offspring pre-natal treatment	1,85	1.38	0.249
	Offspring post-natal treatment	1,85	0.65	0.420
	Sex	1,85	0.41	0.520
	Maternal pre-natal treatment × maternal post-natal treatment	1,85	0.45	0.503
	Offspring pre-natal treatment × offspring post-natal treatment	1,85	3.07	0.080
	Maternal pre-natal treatment × sex	1,85	1,14	0.286
	Maternal post-natal treatment × sex	1,85	1.60	0.206
	Offspring pre-natal treatment × sex	1,85	1.24	0.265
	Offspring post-natal treatment × sex	1,85	0.08	0.777
	Maternal pre-natal treatment × offspring pre-natal treatment	1,85	4.95	**0.026**
	Maternal pre-natal treatment × offspring post-natal treatment	1,85	12.45	**0.0004**
	Maternal post-natal treatment × offspring pre-natal treatment	1,85	0.01	0.927
	Maternal post-natal treatment × offspring post-natal treatment	1,85	0.39	0.530
Latency to enter in zone 2	Maternal pre-natal treatment	1,85	0.13	0.722
	Maternal post-natal treatment	1,85	0.19	0.663
	Offspring pre-natal treatment	1,85	0.17	0.677
	Offspring post-natal treatment	1,85	0.52	0.472
	Sex	1,85	0.54	0.462
	Maternal pre-natal treatment × maternal post-natal treatment	1,85	1.28	0.258
	Offspring pre-natal treatment × offspring post-natal treatment	1,85	0.88	0.348
	Maternal pre-natal treatment × sex	1,85	0.02	0.884
	Maternal post-natal treatment × sex	1,85	0.09	0.765
	Offspring pre-natal treatment × sex	1,85	0.42	0.518
	Offspring post-natal treatment × sex	1,85	0.00	0.945
	Maternal pre-natal treatment × offspring pre-natal treatment	1,85	0.96	0.328
	Maternal pre-natal treatment × offspring post-natal treatment	1,85	0.53	0.468
	Maternal post-natal treatment × offspring pre-natal treatment	1,85	0.17	0.681
	Maternal post-natal treatment × offspring post-natal treatment	1,85	0.16	0.687
Latency to enter in zone 3	Maternal pre-natal treatment	1,85	0.37	0.542
	Maternal post-natal treatment	1,85	0.05	0.821
	Offspring pre-natal treatment	1,85	0.24	0.620
	Offspring post-natal treatment	1,85	0.02	0.893
	Sex	1,85	0.27	0.606
	Maternal pre-natal treatment × maternal post-natal treatment	1,85	0.01	0.920
	Offspring pre-natal treatment × offspring post-natal treatment	1,85	0.00	0.944
	Maternal pre-natal treatment × sex	1,85	0.01	0.927
	Maternal post-natal treatment × sex	1,85	0.01	0.941
	Offspring pre-natal treatment × sex	1,85	0.15	0.697
	Offspring post-natal treatment × sex	1,85	0.07	0.784
	Maternal pre-natal treatment × offspring pre-natal treatment	1,85	0.15	0.697
	Maternal pre-natal treatment × offspring post-natal treatment	1,85	0.01	0.922
	Maternal post-natal treatment × offspring pre-natal treatment	1,85	0.19	0.662
	Maternal post-natal treatment × offspring post-natal treatment	1,85	0.10	0.753
Latency to feed from feeder 1	Maternal pre-natal treatment	1,85	0.27	0.601
	Maternal post-natal treatment	1,85	1.41	0.235
	Offspring pre-natal treatment	1,85	0.06	0.811
	Offspring post-natal treatment	1,85	0.19	0.666
	Sex	1,85	0.14	0.711
	Maternal pre-natal treatment × maternal post-natal treatment	1,85	0.42	0.516
	Offspring pre-natal treatment × offspring post-natal treatment	1,85	0.26	0.611
	Maternal pre-natal treatment × sex	1,85	0.00	0.957
	Maternal post-natal treatment × sex	1,85	0.00	0.947
	Offspring pre-natal treatment × sex	1,85	0.50	0.477
	Offspring post-natal treatment × sex	1,85	0.85	0.357
	Maternal pre-natal treatment × offspring pre-natal treatment	1,85	0.12	0.727
	Maternal pre-natal treatment × offspring post-natal treatment	1,85	1.36	0.243
	Maternal post-natal treatment × offspring pre-natal treatment	1,85	0.30	0.584
	Maternal post-natal treatment × offspring post-natal treatment	1,85	0.03	0.860
Latency to feed from feeder 2	Maternal pre-natal treatment	1,85	0.20	0.654
	Maternal post-natal treatment	1,85	2.39	0.122
	Offspring pre-natal treatment	1,85	0.51	0.476
	Offspring post-natal treatment	1,85	0.01	0.915
	Sex	1,85	0.24	0.626
	Maternal pre-natal treatment × maternal post-natal treatment	1,85	0.18	0.668
	Offspring pre-natal treatment × offspring post-natal treatment	1,85	0.11	0.744
	Maternal pre-natal treatment × sex	1,85	0.12	0.731
	Maternal post-natal treatment × sex	1,85	0.35	0.554
	Offspring pre-natal treatment × sex	1,85	0.06	0.814
	Offspring post-natal treatment × sex	1,85	0.00	0.962
	Maternal pre-natal treatment × offspring pre-natal treatment	1,85	0.24	0.626
	Maternal pre-natal treatment × offspring post-natal treatment	1,85	0.95	0.330
	Maternal post-natal treatment × offspring pre-natal treatment	1,85	0.57	0.448
	Maternal post-natal treatment × offspring post-natal treatment	1,85	0.23	0.628
Latency to feed from feeder 3	Maternal pre-natal treatment	1,85	0.72	0.396
	Maternal post-natal treatment	1,85	0.38	0.536
	Offspring pre-natal treatment	1,85	0.04	0.849
	Offspring post-natal treatment	1,85	0.20	0.653
	Sex	1,85	0.01	0.943
	Maternal pre-natal treatment × maternal post-natal treatment	1,85	0.53	0.468
	Offspring pre-natal treatment × offspring post-natal treatment	1,85	0.21	0.643
	Maternal pre-natal treatment × sex	1,85	0.07	0.793
	Maternal post-natal treatment × sex	1,85	0.01	0.938
	Offspring pre-natal treatment × sex	1,85	0.01	0.937
	Offspring post-natal treatment × sex	1,85	0.07	0.798
	Maternal pre-natal treatment × offspring pre-natal treatment	1,85	0.01	0.934
	Maternal pre-natal treatment × offspring post-natal treatment	1,85	0.00	0.990
	Maternal post-natal treatment × offspring pre-natal treatment	1,85	0.39	0.532
	Maternal post-natal treatment × offspring post-natal treatment	1,85	0.35	0.556
Time spent in the introductory compartment	Maternal pre-natal treatment	1,85	0.21	0.644
	Maternal post-natal treatment	1,85	0.41	0.520
	Offspring pre-natal treatment	1,85	0.06	0.803
	Offspring post-natal treatment	1,85	0.04	0.845
	Sex	1,85	0.86	0.353
	Maternal pre-natal treatment × maternal post-natal treatment	1,85	0.95	0.330
	Offspring pre-natal treatment × offspring post-natal treatment	1,85	2.71	0.100
	Maternal pre-natal treatment × sex	1,85	0.07	0.793
	Maternal post-natal treatment × sex	1,85	0.03	0.861
	Offspring pre-natal treatment × sex	1,85	0.11	0.738
	Offspring post-natal treatment × sex	1,85	0.08	0.781
	Maternal pre-natal treatment × offspring pre-natal treatment	1,85	2.05	0.152
	Maternal pre-natal treatment × offspring post-natal treatment	1,85	1.93	0.165
	Maternal post-natal treatment × offspring pre-natal treatment	1,85	0.17	0.676
	Maternal post-natal treatment × offspring post-natal treatment	1,85	0.30	0.585
Time spent in zone 1	Maternal pre-natal treatment	1,85	1.02	0.312
	Maternal post-natal treatment	1,85	0.25	0.615
	Offspring pre-natal treatment	1,85	0.00	0.982
	Offspring post-natal treatment	1,85	0.07	0.793
	Sex	1,85	0.00	0.969
	Maternal pre-natal treatment × maternal post-natal treatment	1,85	3.12	0.077
	Offspring pre-natal treatment × offspring post-natal treatment	1,85	0.98	0.322
	Maternal pre-natal treatment × sex	1,85	1.23	0.268
	Maternal post-natal treatment × sex	1,85	3.72	0.057
	Offspring pre-natal treatment × sex	1,85	0.31	0.579
	Offspring post-natal treatment × sex	1,85	0.02	0.887
	Maternal pre-natal treatment × offspring pre-natal treatment	1,85	0.04	0.840
	Maternal pre-natal treatment × offspring post-natal treatment	1,85	1.44	0.230
	Maternal post-natal treatment × offspring pre-natal treatment	1,85	0.08	0.775
	Maternal post-natal treatment × offspring post-natal treatment	1,85	1.42	0.234
Time spent in zone 2	Maternal pre-natal treatment	1,85	0.05	0.816
	Maternal post-natal treatment	1,85	0.84	0.359
	Offspring pre-natal treatment	1,85	0.34	0.561
	Offspring post-natal treatment	1,85	0.11	0.741
	Sex	1,85	0.09	0.765
	Maternal pre-natal treatment × maternal post-natal treatment	1,85	0.48	0.488
	Offspring pre-natal treatment × offspring post-natal treatment	1,85	0.21	0.644
	Maternal pre-natal treatment × sex	1,85	0.21	0.647
	Maternal post-natal treatment × sex	1,85	0.01	0.939
	Offspring pre-natal treatment × sex	1,85	3.52	0.058
	Offspring post-natal treatment × sex	1,85	2.03	0.154
	Maternal pre-natal treatment × offspring pre-natal treatment	1,85	0.12	0.726
	Maternal pre-natal treatment × offspring post-natal treatment	1,85	0.03	0.871
	Maternal post-natal treatment × offspring pre-natal treatment	1,85	1.09	0.297
	Maternal post-natal treatment × offspring post-natal treatment	1,85	0.75	0.386
Time spent in zone 3	Maternal pre-natal treatment	1,85	4.82	**0.028**
	Maternal post-natal treatment	1,85	0.64	0.420
	Offspring pre-natal treatment	1,85	2.15	0.140
	Offspring post-natal treatment	1,85	0.22	0.640
	Sex	1,85	0.00	0.960
	Maternal pre-natal treatment × maternal post-natal treatment	1,85	5.38	**0.020**
	Offspring pre-natal treatment × offspring post-natal treatment	1,85	0.17	0.680
	Maternal pre-natal treatment × sex	1,85	1.83	0.177
	Maternal post-natal treatment × sex	1,85	0.49	0.486
	Offspring pre-natal treatment × sex	1,85	1.23	0.310
	Offspring post-natal treatment × sex	1,85	2.51	0.110
	Maternal pre-natal treatment × offspring pre-natal treatment	1,85	1.13	0.280
	Maternal pre-natal treatment × offspring post-natal treatment	1,85	0.06	0.810
	Maternal post-natal treatment × offspring pre-natal treatment	1,85	0.00	0.960
	Maternal post-natal treatment × offspring post-natal treatment	1,85	2.03	0.150
Time spent moving	Maternal pre-natal treatment	1,85	1.87	0.171
	Maternal post-natal treatment	1,85	1.36	0.244
	Offspring pre-natal treatment	1,85	2.37	0.116
	Offspring post-natal treatment	1,85	2.39	0.122
	Sex	1,85	2.29	0.130
	Maternal pre-natal treatment × maternal post-natal treatment	1,85	0.13	0.723
	Offspring pre-natal treatment × offspring post-natal treatment	1,85	3.73	0.055
	Maternal pre-natal treatment × sex	1,85	1.10	0.295
	Maternal post-natal treatment × sex	1,85	1.25	0.263
	Offspring pre-natal treatment × sex	1,85	0.23	0.633
	Offspring post-natal treatment × sex	1,85	1.09	0.297
	Maternal pre-natal treatment × offspring pre-natal treatment	1,85	0.13	0.714
	Maternal pre-natal treatment × offspring post-natal treatment	1,85	2.06	0.151
	Maternal post-natal treatment × offspring pre-natal treatment	1,85	0.72	0.395
	Maternal post-natal treatment × offspring post-natal treatment	1,85	1.13	0.289
Number of feeder visited	Maternal pre-natal treatment	1,85	0.61	0.436
	Maternal post-natal treatment	1,85	1.36	0.243
	Offspring pre-natal treatment	1,85	1.25	0.264
	Offspring post-natal treatment	1,85	0.24	0.623
	Sex	1,85	1.54	0.214
	Maternal pre-natal treatment × maternal post-natal treatment	1,85	0.01	0.927
	Offspring pre-natal treatment × offspring post-natal treatment	1,85	0.65	0.421
	Maternal pre-natal treatment × sex	1,85	0.20	0.652
	Maternal post-natal treatment × sex	1,85	3.34	0.067
	Offspring pre-natal treatment × sex	1,85	1.57	0.210
	Offspring post-natal treatment × sex	1,85	0.02	0.879
	Maternal pre-natal treatment × offspring pre-natal treatment	1,85	0.84	0.359
	Maternal pre-natal treatment × offspring post-natal treatment	1,85	0.13	0.719
	Maternal post-natal treatment × offspring pre-natal treatment	1,85	0.59	0.444
	Maternal post-natal treatment × offspring post-natal treatment	1,85	0.00	0.970
Number of mealworms eaten	Maternal pre-natal treatment	1,85	1.13	0.289
	Maternal post-natal treatment	1,85	1.93	0.180
	Offspring pre-natal treatment	1,85	0.00	0.960
	Offspring post-natal treatment	1,85	6.52	**0.011**
	Sex	1,85	1.14	0.290
	Maternal pre-natal treatment × maternal post-natal treatment	1,85	5.44	**0.020**
	Offspring pre-natal treatment × offspring post-natal treatment	1,85	1.84	0.170
	Maternal pre-natal treatment × sex	1,85	0.02	0.883
	Maternal post-natal treatment × sex	1,85	0.01	0.906
	Offspring pre-natal treatment × sex	1,85	0.42	0.520
	Offspring post-natal treatment × sex	1,85	1.15	0.290
	Maternal pre-natal treatment × offspring pre-natal treatment	1,85	3.12	0.090
	Maternal pre-natal treatment × offspring post-natal treatment	1,85	2.44	0.120
	Maternal post-natal treatment × offspring pre-natal treatment	1,85	0.67	0.410
	Maternal post-natal treatment × offspring post-natal treatment	1,85	0.35	0.550
GR expression hippocampus	Maternal pre-natal treatment	1,85	6.38	**0.012**
	Maternal post-natal treatment	1,85	0.21	0.647
	Offspring pre-natal treatment	1,85	0.20	0.653
	Offspring post-natal treatment	1,85	0.01	0.914
	Sex	1,85	0.10	0.756
	Maternal pre-natal treatment × maternal post-natal treatment	1,85	1.34	0.247
	Offspring pre-natal treatment × offspring post-natal treatment	1,85	0.81	0.367
	Maternal pre-natal treatment × sex	1,85	0.12	0.734
	Maternal post-natal treatment × sex	1,85	0.07	0.787
	Offspring pre-natal treatment × sex	1,85	0.69	0.407
	Offspring post-natal treatment × sex	1,85	2.57	0.054
	Maternal pre-natal treatment × offspring pre-natal treatment	1,85	1.01	0.316
	Maternal pre-natal treatment × offspring post-natal treatment	1,85	1.52	0.218
	Maternal post-natal treatment × offspring pre-natal treatment	1,85	0.24	0.627
	Maternal post-natal treatment × offspring post-natal treatment	1,85	0.59	0.442
MR expression hippocampus	Maternal pre-natal treatment	1,85	0.27	0.607
	Maternal post-natal treatment	1,85	3.80	0.074
	Offspring pre-natal treatment	1,85	0.05	0.822
	Offspring post-natal treatment	1,85	0.48	0.488
	Sex	1,85	1.88	0.170
	Maternal pre-natal treatment × maternal post-natal treatment	1,85	0.73	0.392
	Offspring pre-natal treatment × offspring post-natal treatment	1,85	0.01	0.903
	Maternal pre-natal treatment × sex	1,85	0.00	0.992
	Maternal post-natal treatment × sex	1,85	0.01	0.934
	Offspring pre-natal treatment × sex	1,85	1.21	0.271
	Offspring post-natal treatment × sex	1,85	0.07	0.798
	Maternal pre-natal treatment × offspring pre-natal treatment	1,85	1.45	0.229
	Maternal pre-natal treatment × offspring post-natal treatment	1,85	1.17	0.279
	Maternal post-natal treatment × offspring pre-natal treatment	1,85	0.82	0.364
	Maternal post-natal treatment × offspring post-natal treatment	1,85	1.23	0.267
GR:MR ratio hippocampus	Maternal pre-natal treatment	1,81	11.48	**0.001**
	Maternal post-natal treatment	1,81	1.39	0.213
	Offspring pre-natal treatment	1,81	4.4	**0.036**
	Offspring post-natal treatment	1,81	0.84	0.359
	Sex	1,81	0.79	0.374
	Maternal pre-natal treatment × maternal post-natal treatment	1,81	0.59	0.442
	Offspring pre-natal treatment × offspring post-natal treatment	1,81	0.1	0.748
	Maternal pre-natal treatment × sex	1,81	3.09	0.079
	Maternal post-natal treatment × sex	1,81	0.28	0.595
	Offspring pre-natal treatment × sex	1,81	2.99	0.084
	Offspring post-natal treatment × sex	1,81	3.22	0.081
	Maternal pre-natal treatment × offspring pre-natal treatment	1,81	0.00	0.960
	Maternal pre-natal treatment × offspring post-natal treatment	1,81	2.95	0.086
	Maternal post-natal treatment × offspring pre-natal treatment	1,81	1.82	0.177
	Maternal post-natal treatment × offspring post-natal treatment	1,81	0.44	0.505
GR expression hypothalamus	Maternal pre-natal treatment	1,82	7.68	**0.006**
	Maternal post-natal treatment	1,82	3.21	0.073
	Offspring pre-natal treatment	1,82	6.32	**0.012**
	Offspring post-natal treatment	1,82	0.00	0.990
	Sex	1,82	0.37	0.550
	Maternal pre-natal treatment × maternal post-natal treatment	1,82	0.10	0.750
	Offspring pre-natal treatment × offspring post-natal treatment	1,82	1.63	0.200
	Maternal pre-natal treatment × sex	1,82	0.42	0.519
	Maternal post-natal treatment × sex	1,82	0.67	0.414
	Offspring pre-natal treatment × sex	1,82	0.40	0.530
	Offspring post-natal treatment × sex	1,82	0.04	0.840
	Maternal pre-natal treatment × offspring pre-natal treatment	1,82	0.28	0.600
	Maternal pre-natal treatment × offspring post-natal treatment	1,82	0.15	0.700
	Maternal post-natal treatment × offspring pre-natal treatment	1,82	3.65	0.056
	Maternal post-natal treatment × offspring post-natal treatment	1,82	0.60	0.440
MR expression hypothalamus	Maternal pre-natal treatment	1,82	3.69	**0.049**
	Maternal post-natal treatment	1,82	2.51	0.113
	Offspring pre-natal treatment	1,82	5.08	**0.024**
	Offspring post-natal treatment	1,82	0.32	0.572
	Sex	1,82	0.21	0.648
	Maternal pre-natal treatment × maternal post-natal treatment	1,82	2.04	0.153
	Offspring pre-natal treatment × offspring post-natal treatment	1,82	0.53	0.468
	Maternal pre-natal treatment × sex	1,82	0.00	0.964
	Maternal post-natal treatment × sex	1,82	0.16	0.686
	Offspring pre-natal treatment × sex	1,82	0.10	0.755
	Offspring post-natal treatment × sex	1,82	0.75	0.385
	Maternal pre-natal treatment × offspring pre-natal treatment	1,82	0.00	0.986
	Maternal pre-natal treatment × offspring post-natal treatment	1,82	0.39	0.530
	Maternal post-natal treatment × offspring pre-natal treatment	1,82	0.00	0.975
	Maternal post-natal treatment × offspring post-natal treatment	1,82	1.18	0.278
GR:MR ratio hypothalamus	Maternal pre-natal treatment	1,86	1.80	0.180
	Maternal post-natal treatment	1,78	3.67	0.055
	Offspring pre-natal treatment	1,78	3.37	0.067
	Offspring post-natal treatment	1,78	1.95	0.163
	Sex	1,78	2.66	0.103
	Maternal pre-natal treatment × maternal post-natal treatment	1,78	0.29	0.589
	Offspring pre-natal treatment × offspring post-natal treatment	1,78	0.97	0.324
	Maternal pre-natal treatment × sex	1,78	1.93	0.165
	Maternal post-natal treatment × sex	1,78	3.10	0.085
	Offspring pre-natal treatment × sex	1,78	0.16	0.688
	Offspring post-natal treatment × sex	1,78	3.72	0.052
	Maternal pre-natal treatment × offspring pre-natal treatment	1,78	0.75	0.387
	Maternal pre-natal treatment × offspring post-natal treatment	1,78	0.00	0.975
	Maternal post-natal treatment × offspring pre-natal treatment	1,78	3.82	0.051
	Maternal post-natal treatment × offspring post-natal treatment	1,78	1.17	0.279
GR expression pituitary gland	Maternal pre-natal treatment	1,85	4.40	**0.036**
	Maternal post-natal treatment	1,85	0.26	0.610
	Offspring pre-natal treatment	1,85	0.01	0.940
	Offspring post-natal treatment	1,85	0.23	0.640
	Sex	1,85	0.38	0.540
	Maternal pre-natal treatment × maternal post-natal treatment	1,85	0.02	0.880
	Offspring pre-natal treatment × offspring post-natal treatment	1,85	0.11	0.740
	Maternal pre-natal treatment × sex	1,85	0.00	0.988
	Maternal post-natal treatment × sex	1,85	0.10	0.746
	Offspring pre-natal treatment × sex	1,85	2.48	0.116
	Offspring post-natal treatment × sex	1,85	0.15	0.695
	Maternal pre-natal treatment × offspring pre-natal treatment	1,85	0.31	0.580
	Maternal pre-natal treatment × offspring post-natal treatment	1,85	0.04	0.840
	Maternal post-natal treatment × offspring pre-natal treatment	1,85	0.38	0.540
	Maternal post-natal treatment × offspring post-natal treatment	1,85	0.12	0.730
MR expression pituitary gland	Maternal pre-natal treatment	1,84	4.78	**0.029**
	Maternal post-natal treatment	1,84	0.99	0.320
	Offspring pre-natal treatment	1,84	3.72	0.054
	Offspring post-natal treatment	1,84	1.78	0.183
	Sex	1,84	0.22	0.640
	Maternal pre-natal treatment × maternal post-natal treatment	1,84	0.02	0.888
	Offspring pre-natal treatment × offspring post-natal treatment	1,84	0.23	0.634
	Maternal pre-natal treatment × sex	1,84	1.38	0.240
	Maternal post-natal treatment × sex	1,84	0.18	0.674
	Offspring pre-natal treatment × sex	1,84	0.22	0.637
	Offspring post-natal treatment × sex	1,84	0.11	0.738
	Maternal pre-natal treatment × offspring pre-natal treatment	1,84	2.61	0.106
	Maternal pre-natal treatment × offspring post-natal treatment	1,84	1.05	0.306
	Maternal post-natal treatment × offspring pre-natal treatment	1,84	0.01	0.934
	Maternal post-natal treatment × offspring post-natal treatment	1,84	0.19	0.661
GR:MR ratio pituitary gland	Maternal pre-natal treatment	1,83	2.46	0.120
	Maternal post-natal treatment	1,83	0.36	0.546
	Offspring pre-natal treatment	1,83	0.45	0.504
	Offspring post-natal treatment	1,83	2.05	0.152
	Sex	1,83	0.83	0.361
	Maternal pre-natal treatment × maternal post-natal treatment	1,83	0.83	0.364
	Offspring pre-natal treatment × offspring post-natal treatment	1,83	0.60	0.440
	Maternal pre-natal treatment × sex	1,83	1.03	0.311
	Maternal post-natal treatment × sex	1,83	0.01	0.926
	Offspring pre-natal treatment × sex	1,83	0.14	0.704
	Offspring post-natal treatment × sex	1,83	1.01	0.314
	Maternal pre-natal treatment × offspring pre-natal treatment	1,83	0.19	0.666
	Maternal pre-natal treatment × offspring post-natal treatment	1,83	0.12	0.730
	Maternal post-natal treatment × offspring pre-natal treatment	1,83	0.38	0.537
	Maternal post-natal treatment × offspring post-natal treatment	1,83	3.10	0.078

Significant p-value are indicated in bold.
